# Targeting the Non-Canonical NF-κB Pathway in Chronic Lymphocytic Leukemia and Multiple Myeloma

**DOI:** 10.3390/cancers14061489

**Published:** 2022-03-15

**Authors:** Thomas A. Burley, Emma Kennedy, Georgia Broad, Melanie Boyd, David Li, Timothy Woo, Christopher West, Eleni E. Ladikou, Iona Ashworth, Christopher Fegan, Rosalynd Johnston, Simon Mitchell, Simon P. Mackay, Andrea G. S. Pepper, Chris Pepper

**Affiliations:** 1 Department of Clinical and Experimental Medicine, Brighton and Sussex Medical School, Falmer BN1 9PX, UK; t.burley@bsms.ac.uk (T.A.B.); e.m.kennedy@bsms.ac.uk (E.K.); g.broad1@uni.bsms.ac.uk (G.B.); e.e.ladikou@bsms.ac.uk (E.E.L.); iona.ashworth@nhs.net (I.A.); s.a.mitchell@bsms.ac.uk (S.M.); a.pepper@bsms.ac.uk (A.G.S.P.); 2 Division of Cancer and Genetics, School of Medicine, Cardiff University, Heath Park, Cardiff CF14 4XN, UK; boydm@cardiff.ac.uk (M.B.); lich1@cardiff.ac.uk (D.L.); wootk@cardiff.ac.uk (T.W.); chrisfegan@hotmail.co.uk (C.F.); 3 Strathclyde Institute of Pharmacy and Biomedical Sciences, University of Strathclyde, Glasgow G4 0RE, UK; c.west@beatson.gla.ac.uk (C.W.); simon.mackay@strath.ac.uk (S.P.M.); 4 Drug Discovery Unit, The Beatson Institute for Cancer Research, Garscube Estate, Switchback Road, Bearsden, Glasgow G61 1BD, UK; 5 Department of Haematology, Brighton and Sussex University Hospital Trust, Brighton BN2 5BE, UK; rosalynd.johnston1@nhs.net

**Keywords:** NF-κB inducing kinase, chronic lymphocytic leukemia, multiple myeloma, NF-κB, synergy

## Abstract

**Simple Summary:**

This study was designed to investigate the potential for targeting the NF-κB-inducing kinase, NIK, in two common B-cell malignancies, chronic lymphocytic leukemia (CLL) and multiple myeloma (MM). Using a selective NIK inhibitor, CW15337, we were able to demonstrate that cell lines and primary tumor cells were sensitive to the effects of NIK inhibition, whilst normal lymphocytes were significantly more resistant to its cytotoxic effects. Sensitivity to CW15337 was associated with the nuclear expression of the NF-κB subunit, p52. Importantly, tumor samples from a subset of poor prognosis CLL patients, with mutations in a gene called BIRC3, showed elevated p52 expression and were particularly sensitive to NIK inhibition. Furthermore, the combination of CW15337 and ABT-199 (venetoclax) reversed the drug resistance observed when treating tumor cells with ABT-199 alone. Our study shows the potential for targeting NIK in both CLL and MM.

**Abstract:**

In this study, we evaluated an NF-κB inducing kinase (NIK) inhibitor, CW15337, in primary chronic lymphocytic leukemia (CLL) cells, CLL and multiple myeloma (MM) cell lines and normal B- and T-lymphocytes. Basal NF-κB subunit activity was characterized using an enzyme linked immunosorbent assay (ELISA), and the effects of NIK inhibition were then assessed in terms of cytotoxicity and the expression of nuclear NF-κB subunits following monoculture and co-culture with CD40L-expressing fibroblasts, as a model of the lymphoid niche. CW15337 induced a dose-dependent increase in apoptosis, and nuclear expression of the non-canonical NF-κB subunit, p52, was correlated with sensitivity to CW15337 (*p* = 0.01; r^2^ = 0.39). Co-culture on CD40L-expressing cells induced both canonical and non-canonical subunit expression in nuclear extracts, which promoted in vitro resistance against fludarabine and ABT-199 (venetoclax) but not CW15337. Furthermore, the combination of CW15337 with fludarabine or ABT-199 showed cytotoxic synergy. Mechanistically, CW15337 caused the selective inhibition of non-canonical NF-κB subunits and the transcriptional repression of BCL2L1, BCL2A1 and MCL1 gene transcription. Taken together, these data suggest that the NIK inhibitor, CW15337, exerts its effects via suppression of the non-canonical NF-κB signaling pathway, which reverses BCL2 family-mediated resistance in the context of CD40L stimulation.

## 1. Introduction

Chronic lymphocytic leukemia (CLL) and multiple myeloma (MM) are two of the most frequently diagnosed B-cell malignancies in Europe and North America. Despite the introduction of effective new treatments, they both remain incurable, so there is a clear need to develop alternative therapies. Although CLL and MM represent tumors at different stages of B-cell differentiation, they are both pathologically linked with aberrant NF-κB activation. Indeed, NF-κB has been shown to play a central role in disease progression and drug resistance in these cancers, making it an attractive therapeutic target [1,2]. However, significant safety concerns relating to the use of drugs that target the canonical NF-κB pathway (IKKβ inhibitors) have impeded clinical progress [3]. So, alternative strategies, designed to target specific components of the NF-κB signaling machinery, have the potential to reduce the on-target impact of NF-κB inhibition in normal cells whilst preferentially affecting tumor cells.

Primary MM patient samples and cell lines often show genetic or epigenetic alterations in the NF-κB signaling network, including *NIK*, *TRAF3*, *CYLD*, *BIRC2*, *BIRC3*, *CD40*, *NFKB1* or *NFKB2* [4,5]. In CLL, recurrent mutations have been reported in *NOTCH1*, *BIRC3*, *TRAF3*, *NFΚBIE* and *MYD88* [6]. *NFΚBIE* encodes IκBε, a negative NF-κB regulator. NFΚBIE aberrations have been described in approximately 7% of CLL cases and result in increased nuclear translocation of the NF-KB subunit, RelA [7]. We have previously shown that nuclear DNA binding of RelA is associated with drug resistance and poor clinical outcomes in CLL [2]. *NOTCH1* mutations occur at an even higher frequency in CLL (~12%). Activating mutations in NOTCH1 are associated with poor response to chemotherapy [8] and this may be caused, at least in part, by NOTCH1-mediated NF-κB pathway activation [9,10,11]. *BIRC3* mutations are found in a smaller proportion of CLL patients (~4%) but they impact upon the non-canonical pathway due to the premature truncation of the BIRC3-encoded protein product, cIAP2. This results in the loss of its E3 ubiquitin ligase activity, which is essential for NIK proteasomal degradation. Consequently, NF-κB-inducing kinase (NIK) levels increase leading to the phosphorylation of IKKα, the phosphorylation of p100 and its subsequent processing to p52. This leads to the constitutive activation of non-canonical signaling [12]. Importantly, BIRC3 mutations are associated with a loss of sensitivity to chemotherapy and poor prognosis [13]. Finally, activating mutations in myeloid differentiation primary response gene 88 (MYD88) are found in approximately 3% of CLL cases; these mutations lead to constitutive signaling down-stream of Toll-like receptors (TLRs) and increased expression of the T-cell-attracting chemokines CCL3 and CCL4 [14]. This may be particularly important in the context of the lymphoid niche where T-cell help appears to promote CLL cell proliferation [15]. In MM, Keats et al. [16] reported approximately 20% of mutational events in primary tumors could be mapped to NF-κB signaling. 

In addition to the genetic causes of NF-κB dysregulation in CLL and MM, it is now appreciated that the tumor microenvironment plays a critical role in modulating NF-κB activity in these diseases. In CLL, signaling via the B-cell receptor (BCR), TLRs and CD40, as well as the engagement of the BAFF and APRIL receptors TACI, BAFF-R and BCMA, creates a pro-survival, pro-proliferative niche characterized by NF-κB activation [17,18]. These tissue-derived signals appear to contribute to resistance against both conventional chemotherapies and targeted inhibitors such as ibrutinib and venetoclax [19]. Similarly, in MM, the bone marrow microenvironment promotes MM cell interactions with accessory cells, including bone marrow stromal cells, osteoclasts, osteoblasts and endothelial cells [20,21,22]. These physical interactions activate NF-κB signaling pathways in both malignant and non-malignant cells. In turn, this leads to the secretion of tumor-promoting cytokines and growth factors, which support growth, survival and drug-resistance of MM cells [23,24]. Given the key role of non-canonical NF-κB signaling in the pathology of MM and CLL, here we set out to examine the potential for selectively targeting the non-canonical NF-κB pathway by inhibiting NIK in primary CLL cells and CLL and MM cell lines. We utilized a potent and highly selective NIK inhibitor, called CW15337; the chemical synthesis and characterization of this agent have been described previously [25].

## 2. Materials and Methods

### 2.1. Culture Conditions for Cell Lines, Primary CLL Cells and Normal Lymphocytes

Primary chronic lymphocytic leukemia (CLL) cells (*n* = 15) were obtained from patients attending outpatients’ clinics at the University Hospital of Wales or the Royal Sussex County Hospital with informed consent in accordance with the ethical approval granted by the South East Wales Research Ethics Committee (02/4806) and the Central Bristol Research Ethics Committee (17/SW/0263), respectively. Table 1 shows a summary of the patient characteristics. Age-matched normal B and T cells (*n* = 3) were obtained from healthy volunteers again with informed consent. Four multiple myeloma cell lines, JJN3, U266, RPMI8226 and H929 and the MEC-1 CLL cell line were maintained in liquid culture at densities ranging between 0.5–2 × 10^6^ cells/mL. JJN3 cells were maintained in DMEM medium (Sigma-Aldrich, Gillingham, UK) containing 20% fetal bovine serum (FBS), 1% sodium pyruvate and 1% penicillin and streptomycin. U266, RPMI8226 and H929 cells were maintained in RPMI medium (Merck, Gillingham, UK) containing 20% FBS, 1% L-glutamate and 1% penicillin and streptomycin. All cell lines were purchased from DSMZ (Braunschweig, Germany) and were used for these experiments within 6 months of purchase. In each case, the provenance of the cell lines was verified by multiplex PCR of minisatellite markers, and all were regularly confirmed to be mycoplasma-free by PCR. In terms of NF-κB mutations, JJN3 cells possess an EFTUD2-NIK fusion gene which lacks the TRAF3 binding domain resulting in the accumulation of a cytoplasmic EFTUD2-NIK fusion protein. U266 and RPMI8226 cells exhibit a TRAF3 mutation resulting in the stabilization of wild-type NIK protein. H929 cells have no known NF-κB-related mutations [4].

### 2.2. Cell Separation and Counting

Primary CLL and normal lymphocytes were isolated by means of density gradient centrifugation using Histopaque (Sigma-Aldrich) and were then maintained in RPMI medium containing 10% FBS, 5 ng/mL IL-4, 1% L-glutamine and 1% penicillin and streptomycin. All cells were cultured at 37 °C in 5% CO_2_ atmospheric conditions. Cell counts and viability (trypan blue exclusion) were determined using the Vi-Cell XR cell counter (Beckman Coulter, High Wycombe, UK) or a Countess II cell counter (ThermoFisher, East Grinstead, UK).

### 2.3. CD40L Co-Culture Conditions

Primary CLL cells were co-cultured on CD40L-expressing 3T3 fibroblasts (10:1 ratio) to mimic the lymph node microenvironment. Subsequently, synergy between CW15337 and ABT-199 or fludarabine was determined under these cytoprotective conditions. Briefly, NIH/3T3 murine fibroblasts transfected with human CD40L or untransfected (NTL) 3T3 cells were seeded at 10^5^/mL in 24-well plates in RPMI-1640 complete medium and incubated overnight to allow cells to adhere. The next day, 1 × 10^6^ peripheral blood mononuclear cells (PBMCs) from CLL patients, MEC-1 cells or RPMI8226 cells were cultured in monoculture, on NTL cells or on CD40L-expressing 3T3 cells as previously described [26]. Cells were then harvested after 8 h of culture. Subsequently, the expression of markers associated with cellular activation (Ki-67, HLA-DR- and CD69) was quantified on CD19+/CD5+ CLL cells in the PBMC samples and on CD19+ MEC-1 cells using mean fluorescent intensity (MFI) values. Staining with fluorescence-labeled antibodies was carried out according to the antibody manufacturer’s instructions (Biolegend, London, UK) followed by the acquisition of 10,000 events on a CytoFLEX LX flow cytometer (Beckman Coulter).

### 2.4. Flow Cytometric Quantification of NF-κB p52

NF-κB p52 expression levels were quantified using a phycoerythrin-labeled anti-NF-κB p52 (Santa Cruz, Heidelberg, Germany). Briefly, aliquots of 1 × 10^6^ primary CLL cells were treated with or without CW15337 (0–5 μM) for 24 h. Subsequently, cells were labeled with CD5-FITC and CD19-APC prior to being fixed and permeabilized using fix/perm reagent (Biolegend). Then, 5 μL of anti-NF-κB p52 antibody was added to each aliquot of cells. After incubation for 10 min, cells were washed and then resuspended in 1% paraformaldehyde solution prior to being analyzed by flow cytometry using a CytoFLEX LX flow cytometer.

### 2.5. Reagents

CW15337 was synthesized, purified and characterized as previously reported [25]. Fludarabine and ABT-199 (venetoclax) were purchased from Selleckchem, Houston, TX, USA).

### 2.6. Measurement of In Vitro Apoptosis

Aliquots of each cell type (1 × 10^6^ cells) were cultured for 48 h, harvested by centrifugation (300× *g* for 5 min) and then resuspended in 195 µL of a calcium-rich buffer (Biolegend). Subsequently, 5 µL of Annexin V (Biolegend) was added to the cell suspension, and cells were incubated in the dark for 10 min prior to washing. Cells were finally resuspended in 190 µL of calcium-rich buffer together with 10 µL of propidium iodide (PI). Apoptosis was assessed by dual-colour immunofluorescent flow cytometry using an Accuri C6 flow cytometer, and data were analyzed using CFlow software (BD Biosciences, San Jose, CA, USA).

### 2.7. Measurement of Apoptosis in Normal B- and T-Lymphocytes

Peripheral blood mononuclear cells from age-matched healthy donors (1 × 10^6^ cells) were treated with concentrations of CW15337 between 0.1 and 10 μM for 48 h. Cells were then harvested and stained with FITC-conjugated Annexin V and 7-AAD (Biolegend). Apoptosis was quantified by flow cytometry by the summation of the Annexin V^+^/7-AAD^-^ and Annexin V^+^/7-AAD^+^ quadrant gates.

### 2.8. Proliferation and Cell Cycle Analysis

Cell proliferation was assessed using a combination of trypan blue staining and volumetric cell counting, using a Countess II cell counter (Thermofisher). Cell cycle analysis was performed on cells that were fixed in ice-cold ethanol and then labeled with a propidium iodide solution (50 μg/mL) containing RNase A (100 μg/mL). Cells were analyzed using an Accuri C6 flow cytometer with CFlow software, and 10,000 events were acquired. Subsequently, the cell cycle distribution of each sample was quantified using the FlowJo cell cycle analysis module.

### 2.9. Enzyme Linked Immuno-Sorbent Assay (ELISA) for NF-κB Subunits

Primary CLL cells and myeloma and CLL cell lines were initially assessed for the basal expression of NF-κB subunits. Subsequently, RPMI8226 cells, MEC-1 cells and primary CLL cells were treated for 4 h with CW15337 (0 μM–5 μM), either in monoculture or co-culture on CD40L-expressing 3T3 cells. Pellets containing 5 × 10^6^ cells were then harvested, and subsequently, nuclear extracts were prepared using a nuclear extraction kit (Active Motif, Waterloo, Belgium). Total protein was determined by DC protein assay (Bio-Rad, Watford, UK) in each nuclear extract using a standard curve of known concentrations of BSA. Nuclear extracts containing 1 μg of total protein from each treatment were then added to an NF-κB family kit (Active Motif) in accordance with the manufacturer’s instructions. Levels of p65/RelA, p50, p52, RelB and c-Rel DNA binding were then assessed to determine relative levels of each subunit in the nuclear extracts. The absorbance readings at 450 nm are directly proportional to the amount of each respective NF-κB protein bound to the NF-κB consensus sequences.

### 2.10. Transwell Migration Assay

To evaluate MEC-1 cell migration, transwell plates with a filter diameter of 6.5 mm and pore size of 5.0 μm were used. Cells were diluted to 1 × 10^6^ cells/mL in RPMI complete medium, with or without the addition of CW15337 (0, 2.5, 5, 10 μM). Subsequently, 5 × 10^5^ cells/well were seeded into the upper transwell insert. The lower compartment of the chamber was filled with 500 μL of complete medium supplemented with 100 ng/mL CXCL12. The chambers were incubated at 37 °C for 24 h and then removed from the plate. Cells that had migrated to the lower chamber were harvested and counted using a CytoFLEX LX flow cytometer. The level of apoptosis induced by CW15337 during the assay was quantified by harvesting cells from the upper chamber of the transwell (unmigrated cells), followed by labeling with Annexin V and 7-AAD (as above).

### 2.11. Synergy between CW15337 and ABT-199 or Fludarabine

The synergy between CW15337 in combination with either the BCL2 antagonist, ABT-199 or fludarabine was determined in primary CLL cells (*n* = 6). The molar ratios were experimentally determined using the mean LD_50_ value for CW15337 and clinically achievable concentrations of ABT-199 and fludarabine. The fixed molar ratio for CW15337:ABT-199 was 100:1, and it was 1:1 for CW15337:fludarabine. Cells were treated with each drug individually and in combination at the defined molar ratio. Treated and untreated cells were co-cultured on CD40L-expressing 3T3 cells for 48 h, before being labeled with Annexin V FITC/7-AAD and then analyzed on an Accuri C6 flow cytometer. The expected drug combination responses were calculated based on the BLISS reference model using SynergyFinder (https://synergyfinder.fimm.fi, accessed on 30 January 2022) [27].

### 2.12. RNA Isolation

Primary CLL cells were co-cultured on and off CD40L-expressing 3T3 fibroblasts for 4 h in triplicate. From each sample, 5 × 10^6^ cells were then harvested, washed in ice cold PBS and re-suspended in 1 mL of Trizol reagent (ThermoFisher). RNA was extracted following the addition of chloroform and 70% ethanol, and a RNeasy micro kit (Qiagen, Manchester, UK) was then used in accordance with the manufacturer’s instructions to isolate RNA to be used in RNA sequencing (RNA-seq) analysis. The data generated in this publication will be deposited in NCBI’s Gene Expression Omnibus and will be accessible through GEO Series accession number GSE198456 (https://www.ncbi.nlm.nih.gov/geo/query/acc.cgi?acc=GSE198456, accessed on 30 January 2022).

### 2.13. RNA-Sequencing and Analysis

RNA was extracted using the RNeasy Micro Kit (Qiagen) as per the manufacturer’s instructions. Strand-specific RNA-seq libraries were prepared from the CLL cell paired samples using the NEBNext^®^ Ultra™ RNA Library Prep Kit for Illumina^®^ according to manufacturer’s instructions and paired end sequenced (2 × 75 cycles) using the Illumina NextSeq500 (Illumina, San Diego, CA, USA). Approximately 20 million sequencing read pairs were obtained per sample. Transcripts in the human genome (hg-38) were quantified from the paired-end reads using Kallisto software and gene-level count data processed and analyzed for differential expression using DESeq2. XenofilteR was utilized to remove any mouse sequence reads from the co-culture experiment. Over-representation analysis (ORA) was performed using WebGestalt. Signalling pathway networks displaying the differential expression data were created using Cytoscape v3.7.2.

### 2.14. Reverse Transcription of RNA

RNA was extracted as described above and was quantified using the NanoDrop 1000 spectrophotometer (Thermo Fisher Scientific, Waltham, MA, USA). One microgram of RNA was converted to cDNA using High-capacity cDNA Reverse Transcription Kit (Applied Biosystems, San Francisco, CA, USA). For each reaction, the following were mixed: 2 μL 10 × RT buffer, 0.8 μL 25 × dNTP mix (100 mM), 2 μL 10 × RT random primers, 1 μL of reverse transcriptase. The mixture was topped up with respective amount of nuclease-free water to 20 μL. The protocol was as follows: 25 °C for 10 min, 37 °C for 120 min, 85 °C for 5 min, and 4 °C holding temperature.

### 2.15. Quantitative Real-Time PCR (QPCR)

QPCR was performed on a Quant Studio Real-Time PCR Detection System with SYBR Green Supermix (Bio-Rad). For 1 QPCR reaction, the following were mixed: 5 μL of SYBR Green Supermix, 300 nM of forward primer, 300 nM of reverse primer, 1.9 μL of nuclease-free water, 2.5 μL of cDNA (2.5 ng/μL), to a final volume of 10 μL. The samples were loaded in duplicate on a 384-well plate (Applied Biosystems). The thermal cycling conditions were as follows: 95 °C for 3 min, 95 °C for 5 s, 60 °C for 30 s, repeated for 39 cycles and then 65 °C for 30 s and 4 °C holding temperature. Fold changes were normalized to β-actin gene expression and were based on relative expression values calculated using the 2^−ΔΔC(T)^ method.

β-Actin primers: Forward sequence, CACCATTGGCAATGAGCGGTTC; reverse sequence, AGGTCTTTGCGGATGTCCACGT.
BCL2L1 primers: Forward sequence, GCCACTTACCTGAATGACCACC; reverse sequence, AACCAGCGGTTGAAGCGTTCCT.
BCL2A1 primers: Forward sequence, GGATAAGGCAAAACGGAGGCTG; reverse sequence, CAGTATTGCTTCAGGAGAGATAGC.
MCL1 primers: Forward sequence, CCAAGAAAGCTGCATCGAACCAT; reverse sequence, CAGCACATTCCTGATGCCACCT.

### 2.16. Statistical Analysis

All statistical analysis was performed using Graphpad Prism 9.0 (Graphpad Software, San Diego, CA, USA). In all cases, the normal distribution of the data was assessed using the omnibus K2 test. Univariate comparisons were made using Student’s *t*-test for paired and unpaired observations or a two-way ANOVA with Dunnett’s correction for multiple comparisons. All toxicity data from drug treatment were used to produce sigmoidal dose–response curves from which LD_50_ values were calculated. Toxicity data from synergy experiments were processed using SynergyFinder (https://synergyfinder.fimm.fi, accessed on 30 January 2022) with positive and negative values denoting synergy and antagonism, respectively. BLISS scores > 10 strongly suggest synergistic interactions.

## 3. Results

### 3.1. Evaluation of the Effects If the NIK Inhibitor, CW15337

**Figure 1:**
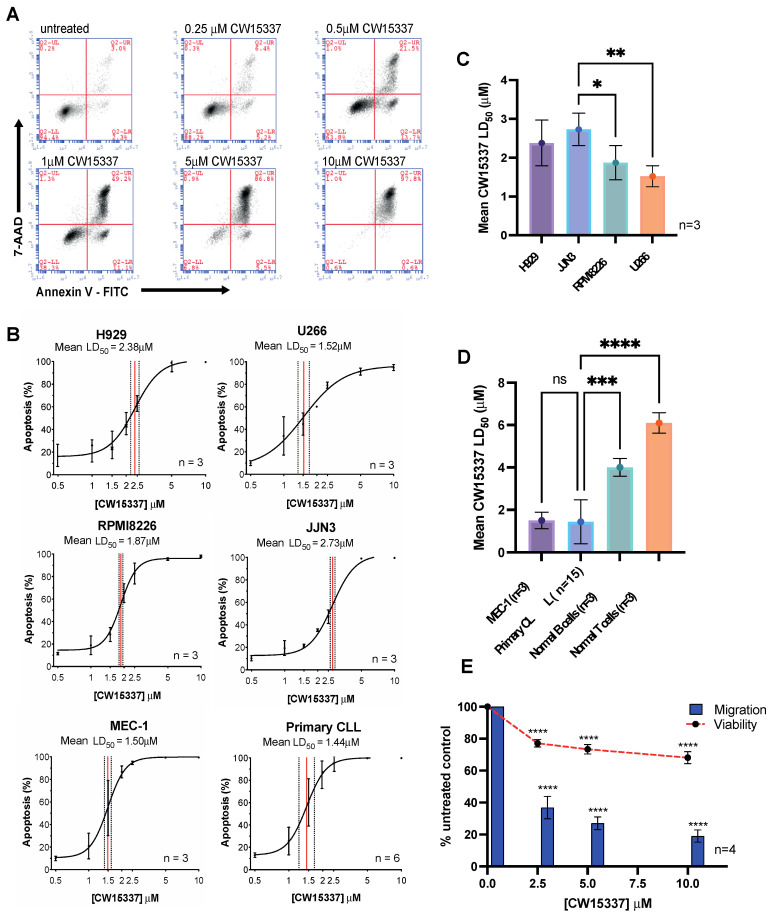
Primary CLL cells and CLL and myeloma cell lines are all susceptible to NF-κB-inducing kinase (NIK) inhibition. (**A**) An example of Annexin V and 7-AAD bivariate plots obtained from primary CLL cells treated with increasing concentrations of CW15337. A concentration-dependent increase in the proportion of Annexin V^+^/7-AAD^−^ and Annexin V^+^/7-AAD^+^ was observed. (**B**) Sigmoidal dose–response curves illustrating the comparative effects of CW15337 on the H929, U266, RPMI8226, JJN3, MEC-1 cell lines and primary CLL cells. (**C**) Comparative analysis of the mean LD_50_ values for CW15337 in the four multiple myeloma cell lines revealed heterogeneous sensitivity to the effects of CW15337 between the cell lines. (**D**) CW15337 was significantly more potent in MEC-1 cells and primary CLL cells when compared with normal B- and T-lymphocytes. (**E**) CW15337 inhibited the migration of MEC-1 cells in a concentration-dependent manner. **** *p* < 0.0001, *** *p* < 0.001, ** *p* < 0.01 and * *p* < 0.05. ns = not significant.

Initial experiments focused on establishing the relative cytotoxicity of the NIK inhibitor, CW15337, in primary CLL cells, CLL and MM cell lines and normal B- and T-lymphocytes. In all cases, CW15337 induced dose-dependent apoptosis, quantified using Annexin V and 7-AAD labeling (Figure 1A). All the malignant cell types evaluated showed low micromolar LD_50_ values with distinct responses between cell lines and heterogeneity between the primary CLL samples (Figure 1A–C). Normal B- and T-lymphocytes were significantly less sensitive to the cytotoxic effects of CW15337 (Figure 1D), consistent with the low levels of nuclear NF-κB and low expression of NIK, commonly found in unstimulated lymphocytes [28,29]. In addition to its cytotoxic effects, CW15337 also significantly inhibited MEC-1 cell migration, in 24 h transwell assays, in a concentration-dependent manner (Figure 1E). The anti-migratory effect could not be explained by CW15337-induced apoptosis over the same timeframe (Figure 1E).

### 3.2. Comparative Sensitivity to CW15337 in CLL Prognostic Subsets

**Figure 2:**
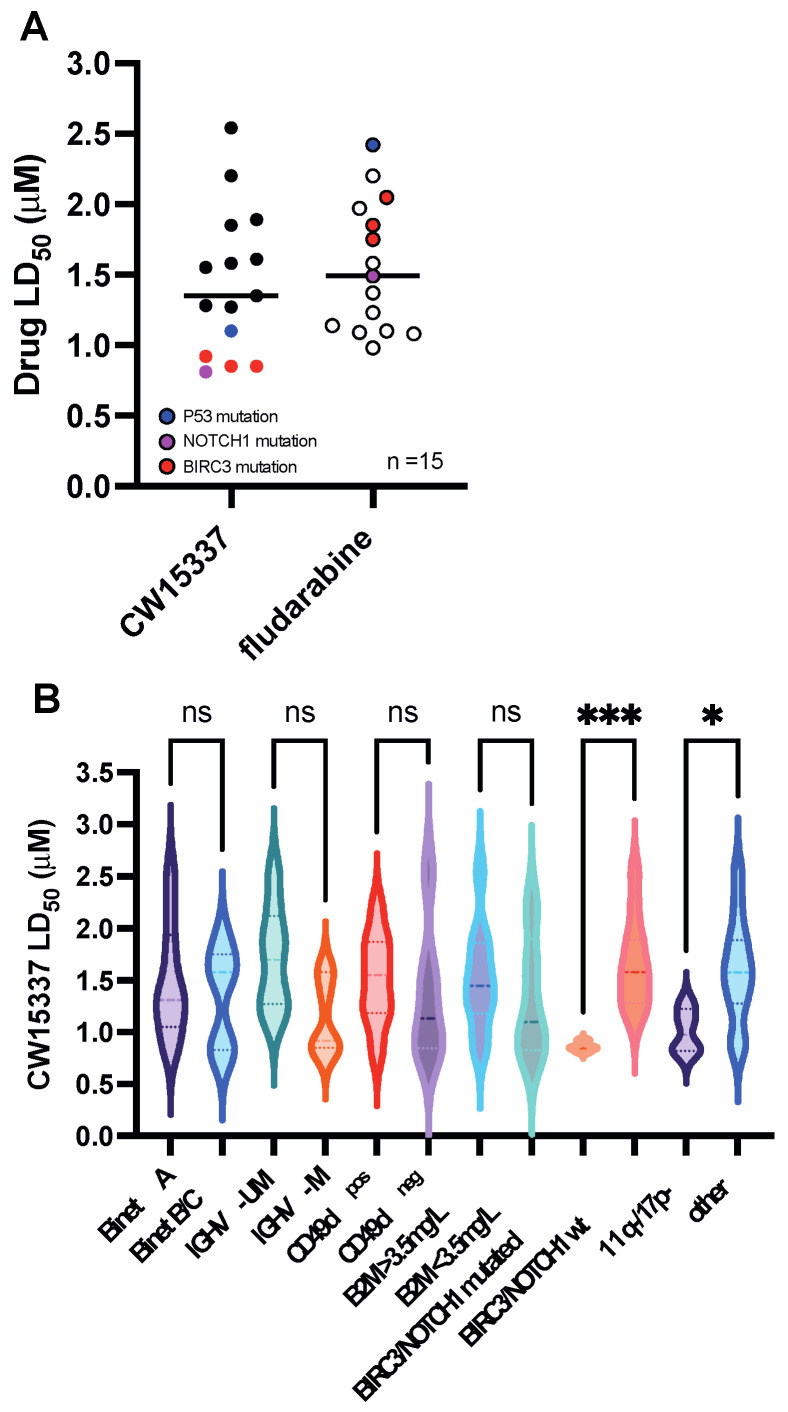
CW15337 is preferentially cytotoxic in CLL cells that carry a BIRC3 or NOTCH1 mutation. (**A**) All of the CLL samples tested had low micromolar LD_50_ values, with a mean of 1.44 μM for the entire cohort. (**B**) Comparative analysis of the sensitivity to CW15337 in CLL prognostic subsets revealed that samples derived from patients carrying a BIRC3 or NOTCH1 mutation were particularly sensitive to CW15337; the same samples showed increased in vitro resistance to fludarabine. *** *p* < 0.001, and * *p* < 0.05. ns = not significant.

All of the CLL samples treated with CW15337 (*n* = 15) showed low micromolar LD_50_ values, with a mean LD_50_ value for the cohort of 1.44 μM (Figure 2A). Although the total number of individual CLL samples analyzed was relatively small, we next examined whether sensitivity to CW15337 was associated with any of the known CLL prognostic markers (see Table 1). There was no significant difference in mean LD_50_ value between samples derived from Binet stage A and Binet stage B/C patients, *IGHV*-mutated and *IGHV*-unmutated samples, CD49d-positive and CD49d-negative samples (≥/<30%) or samples with high or low β2-microglobulin concentrations (≥/<3.5 mg/L) (Figure 2B). Notably, primary CLL samples derived from patients carrying BIRC3 mutations (*n* = 3) or NOTCH1 mutations (*n* = 1) in their CLL tumor cells were particularly sensitive to NIK inhibition (Figure 2A). In keeping with previous reports, the BIRC3 mutated samples showed increased in vitro resistance to fludarabine (Figure 2A) [13,30].

### 3.3. Basal Nuclear p52 Expression Predicts for an In Vitro Response to CW15337

**Figure 3:**
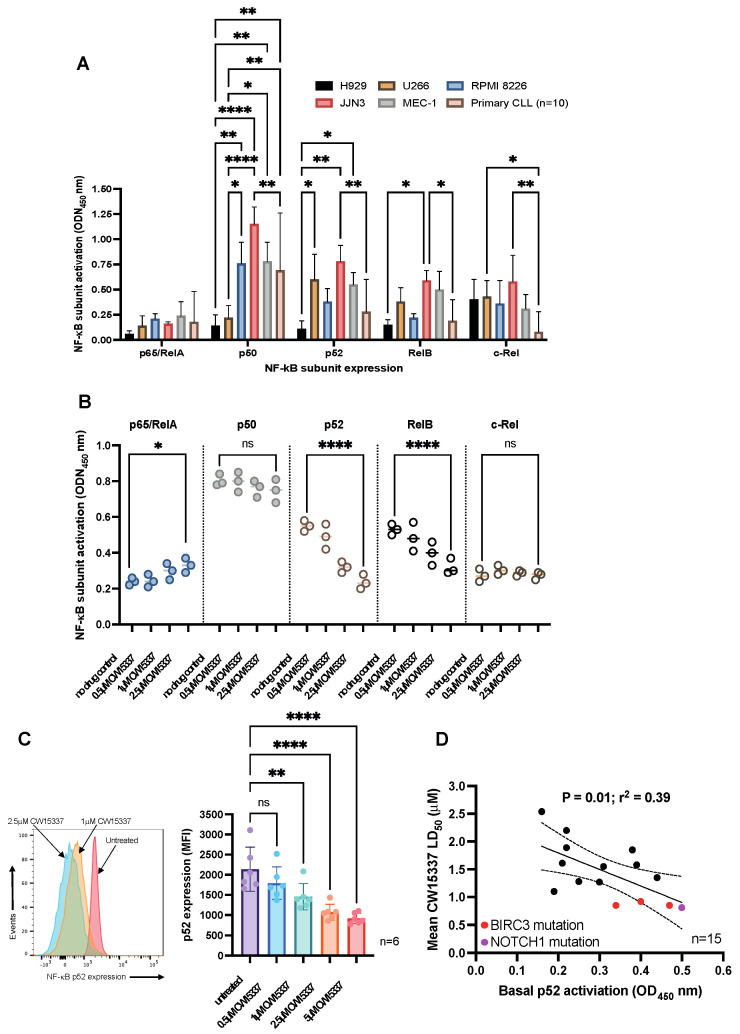
CW15337 preferentially inhibits the nuclear expression of p52 and RelB NF-κB subunits. (**A**) Each cell line evaluated showed a distinct pattern of constitutive NF-κB subunit activation in nuclear extracts. (**B**) Exposure to CW15337 resulted in a concentration-dependent reduction in two non-canonical NF-κB subunits, p52 and RelB. (**C**) MEC-1 cells treated with increasing concentrations of CW15337 showed a marked inhibition of p100 processing to p52. (**D**) Constitutive p52 expression in nuclear extracts from primary CLL samples (*n* = 15) were correlated with in vitro sensitivity to CW15337. **** *p* < 0.0001, ** *p* < 0.01 and * *p* < 0.05. ns = not significant.

To investigate the molecular mechanisms that underpin the cytotoxic effects of CW15337, we next quantified the basal nuclear activity of NF-κB subunits in nuclear extracts from the cell lines and primary CLL patient samples (*n* = 15). We found marked variation in the relative nuclear abundance of each NF-κB subunit between the samples, which reflects their differential activation of canonical and non-canonical signaling governed, to some extent, by their different genetic backgrounds (Figure 3A). The differences in NF-κB subunit activity in each cell line suggests that differential activation of canonical and non-canonical signaling may be governed, to some extent, by differential genetic backgrounds. For example, the H929 cell line has no reported mutations in NF-κB-associated genes and showed the lowest expression of NF-κB subunits. In contrast, U266 and RPMI8226 cells possess TRAF3 mutations, which result in NIK stabilization and the processing of p100 to p52. This is reflected in the relative abundance of p52 in the nuclear extracts from each of these cell lines. We went on to investigate the consequence of NIK inhibition on NF-κB subunit expression in MEC-1 cells. Treatment of MEC-1 cells with CW15337 for 4 h resulted in a small, but significant, increase in p65/RelA (*p* < 0.05) and a significant, dose-dependent, reduction in both p52 (*p* < 0.0001) and RelB (*p* < 0.0001; Figure 3B). Consistent with these findings, flow cytometric analysis of the NF-κB subunit, p52, demonstrated that CW15337 significantly reduced the level of p52 in primary CLL cells in a concentration-dependent manner (Figure 3C). Furthermore, basal activity of p52 in primary CLL cells (as measured by ELISA) correlated with sensitivity to CW15337 (Figure 3D), suggesting that cells with higher nuclear p52, including those carrying NOTCH1 or BIRC3 mutations, are more sensitive to NIK inhibition.

### 3.4. The Pro-Proliferative Effects of CD40L Co-Culture Are Reversed by NIK Inhibition

**Figure 4:**
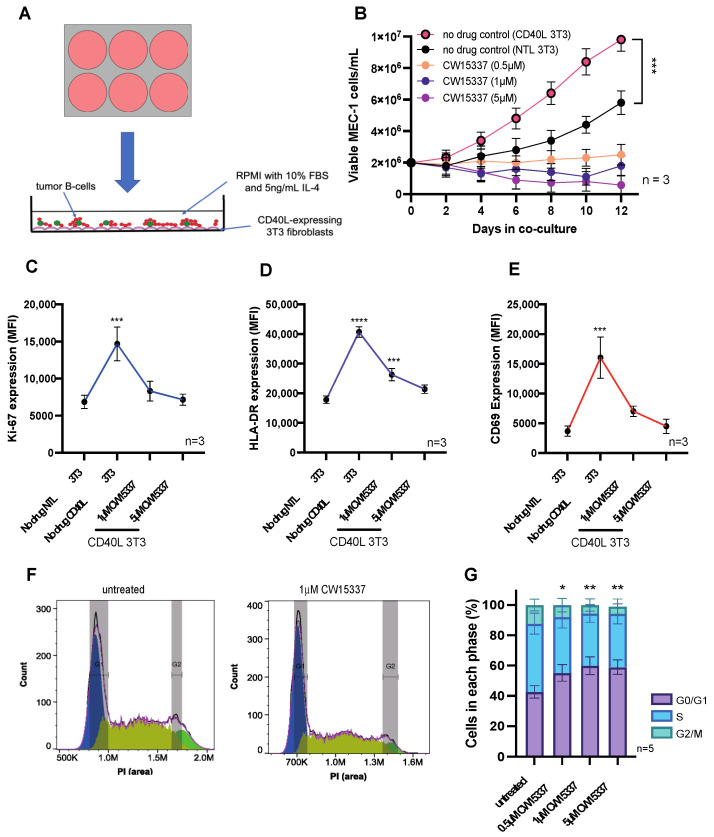
Co-culture on CD40L-expressing 3T3 cells drives MEC-1 cell activation and proliferation, which was reversed by the addition of CW15337. (**A**) Untransfected NIH/3T3 cells or NIH/3T3 cells transfected with human CD40L were plated prior to the addition of malignant B cells (1:10 ratio). (**B**) MEC-1 cells co-cultured on CD40L 3T3 cells showed significantly increased cell growth when compared to those cultured on untransfected 3T3 cells. MEC-1 cell growth was abolished by the addition of CW15337 to the CD40L 3T3 co-cultures. (**C**–**E**) In keeping with these findings, MEC-1 cells showed a marked increase in Ki67, HLA-DR and CD69 when co-cultured on CD40L 3T3 cells, which was reversed by the addition of CW15337. (**F**,**G**) CW15337 induced a concentration-dependent G1 arrest in MEC-1 cells co-cultured on CD40L 3T3 cells. **** *p* < 0.0001, *** *p* < 0.001, ** *p* < 0.01 and * *p* < 0.05.

To model the lymph node microenvironment of CLL, we used a well-established in vitro co-culture system utilizing NIH/3T3 mouse fibroblasts [26,31,32] were transfected with human CD40L to mimic interactions with activated T-cells that occur in the lymphoid tissues (Figure 4A). We showed that this co-culture system induced the proliferation and survival of MEC-1 cells. The addition of CW15337 into the cultures inhibited the growth of MEC-1 cells, which was maintained throughout the time course of the study (Figure 4B). Consistent with these findings, proliferation (Ki-67) and cellular activation markers (HLA-DR and CD69) were shown to be induced by co-culture with CD40L 3T3 cells; all these markers were inhibited in a concentration-dependent manner by CW15337 (Figure 4C–E); expression was repressed to similar levels in MEC-1 cells co-cultured on untransfected 3T3 cells. Furthermore, CW15337 induced G1 cell cycle arrest in MEC-1 cells, again in a dose-dependent fashion (Figure 4F,G).

### 3.5. CD40L 3T3 Co-Culture Induced Both Canonical and Non-Canonical NF-κB Activation

**Figure 5:**
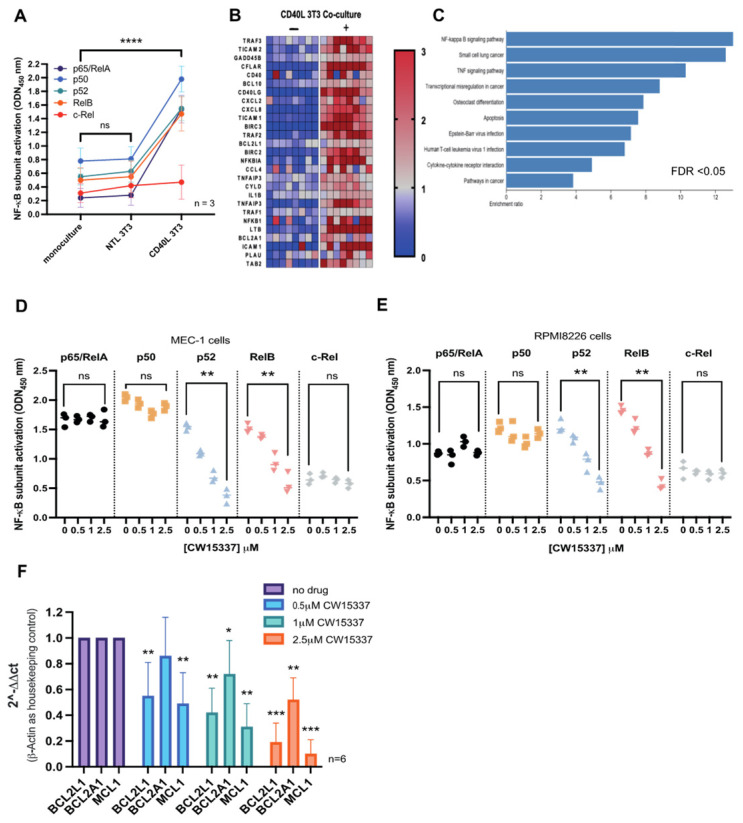
CW15377 preferentially inhibits the non-canonical NF-κB pathway in tumor cells co-cultured on CD40L 3T3 cells. (**A**) Nuclear extracts were prepared from primary CLL cells following monoculture, co-culture on untransfected 3T3 cells and CD40L 3T3 cells. Extracts from CLL cells co-cultured on CD40L 3T3 cells showed a marked increase in both canonical and non-canonical subunit. (**B**,**C**) RNA sequencing showed a marked transcriptional activation of known NF-κB-target genes. Furthermore, NF-κB regulated genes were significantly overrepresented in the differentially expressed gene list. CD40L 3T3 co-culture of (**D**) MEC-1 cells and (**E**) RPMI8226 cells for 24 h with or without the addition of CW15337 for the last 8 h, showed that CW15337 preferentially inhibited the nuclear expression of p52 and RelB in a concentration-dependent manner. (**F**) QRT-PCR analysis of primary CLL cells (*n* = 6) confirmed that the addition of CW15337 could repress the transcriptional activation of BCL2L1, MCL1 and to a lesser extent, BCL2A1 induced by CD40L 3T3 co-culture. FDR = false discovery rate. **** *p* < 0.0001, *** *p* < 0.001, ** *p* < 0.01 and * *p* < 0.05. ns = not significant.

We next explored the consequences of CD40L 3T3 co-culture on NF-κB subunit nuclear localization and global gene expression in primary CLL cells (*n* = 3). Co-culture on CD40L 3T3 cells markedly induced the nuclear expression of all NF-κB subunits, except for c-Rel (Figure 5A). RNA-sequencing of CLL cells following 4 h on CD40L 3T3 co-culture confirmed significant over-representation of NF-κB target genes in the differentially expressed gene list, with NF-κB signaling pathways being the most enriched transcriptional signature (Figure 5B,C). Subsequently, nuclear extracts were generated from MEC-1 cells and RPMI8226 cells after 24 h on CD40L 3T3 co-culture with and without the addition of CW15337 for the final 8 h. Both cell lines showed concentration-dependent inhibition of both p52 and RelB (Figure 5D,E). CW15337 was found to be highly selective for RelB and p52, while as canonical NF-κB components, RelA, p50 and c-Rel were unaffected (Figure 5D,E). Primary CLL samples (*n* = 5) were subjected to the same co-culture conditions, with and without the addition of CW15337 for the final 8 h. Co-culture significantly increased the relative transcription of many NF-κB-regulated genes (Figure 5B), including three BCL2-family anti-apoptotic genes, BCL2L1 (11.3-fold), BCL2A1 (11.3-fold) and MCL1 (1.5-fold). QRT-PCR analysis revealed that the expression of all three genes was significantly repressed following treatment with CW15337, when normalized to β-Actin (Figure 5F), indicating that they are all regulated by the non-canonical NF-κB pathway.

### 3.6. CW15337 Synergises with ABT-199 and Fludarabine under CD40L Co-Culture Conditions

**Figure 6:**
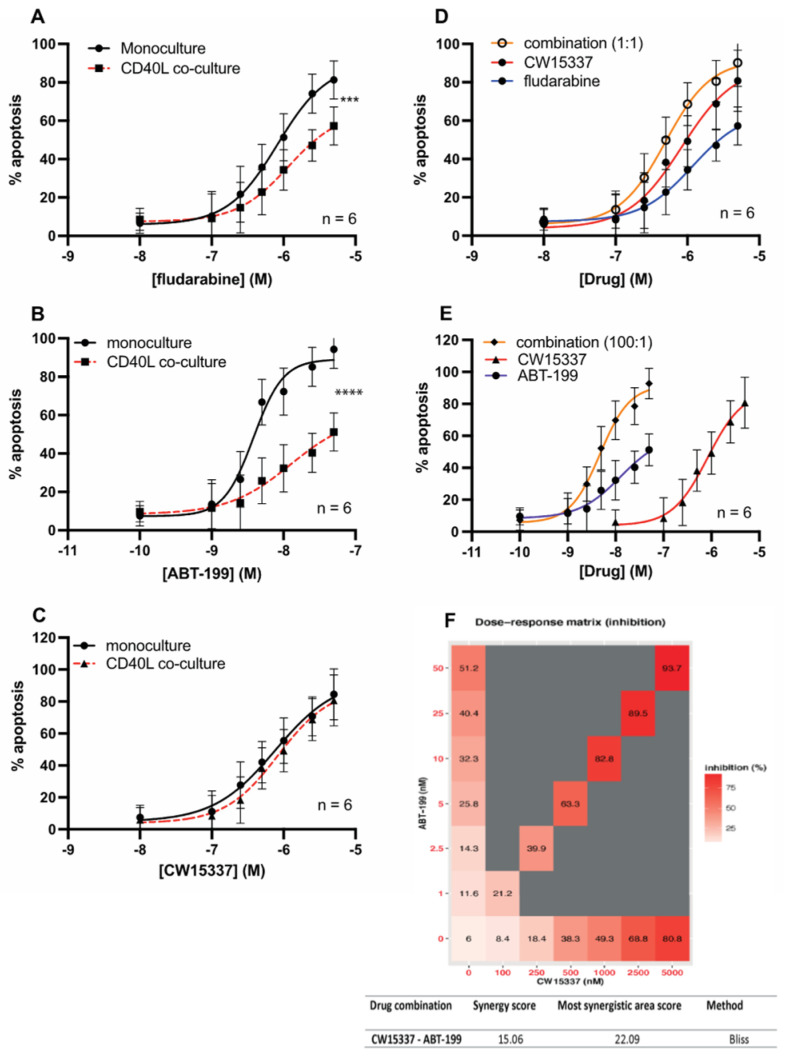
CW15337 is synergistic with fludarabine and ABT-199 in the setting of CD40L 3T3 co-culture. Primary CLL cells showed a marked increase in resistance to (**A**) fludarabine and (**B**) ABT-199 when co-cultured on CD40L 3T3 cells. (**C**) In contrast, the sensitivity to CW15337 was not significantly affected by CD40L co-culture. The combination of CW15337 with (**D**) fludarabine and (**E**) ABT-199 showed synergistic interactions. (**F**) The combination of CW15337 and ABT-199 (100:1) was shown to be particularly synergistic under CD40L co-culture conditions, using SynergyFinder software (https://synergyfinder.fimm.fi, accessed on 30 January 2022). BLISS scores ≥ 10 indicate synergy, the average synergy score of CW15337 and ABT-199 was 15.06. **** *p* < 0.0001, *** *p* < 0.001.

In keeping with previous studies [31,32], here we demonstrated that CD40L stimulation of CLL cells results in increased NF-κB signaling (both canonical and non-canonical) and the transcriptional activation of NF-κB target genes, including the anti-apoptotic genes of the BCL2 family, BCL2L1, BCL2A1 and MCL1. Clinically, CD40L stimulation takes place in the lymphoid tissues in CLL and in the bone marrow niche in MM, and this leads to the emergence of drug resistance to a variety of therapeutics including fludarabine, bortezomib and venetoclax (ABT-199) [21,32,33]. Given that CW15337 was able to reverse non-canonical NF-κB activation and thereby repress the transcription of BCL2L1, BCL2A1 and MCL1, we set out to establish whether CW15337 could reverse CD40L-induced drug resistance. As expected, co-culture of primary CLL cells on CD40L-expressing 3T3 cells caused a marked increase in resistance to both fludarabine (Figure 6A) and ABT-199 (Figure 6B). In contrast, CW15337 retained its activity under co-culture conditions (Figure 6C). The combination of CW15337 with fludarabine (Figure 6D) or ABT-199 (Figure 6E) showed synergistic interactions, which overcame the co-culture-induced resistance. Figure 6F shows the combination of CW15337 and ABT-199

## 4. Discussion

Although the treatment of CLL and MM patients has undergone a revolution in recent years, both conditions remain incurable and this is caused, at least in part, by the emergence of acquired drug resistance. Although drug resistance can arise through a variety of different mechanisms, the activation of NF-κB has also been implicated in the development of chemotherapeutic drug resistance in myeloma and CLL [34,35,36]. DNA damaging agents, including fludarabine and melphalan, increase the activity of NF-κB in residual tumor cells, rendering them more resistant to the cytotoxic effects of these treatments [2,37]. More recently, NF-κB has also been implicated in the development of resistance to bortezomib, ibrutinib and venetoclax [19,38].

Ample evidence exists to support the rationale of pursuing novel therapies which target NF-κB. However, due to its critical role in a wide spectrum of cellular processes and the lack of clarity around which of the various NF-κB subunits orchestrate essential cellular functions, direct pharmacological targeting of NF-κB has so far failed due to concerns over potential on-target effects and associated toxicities, primarily through the inhibition of the canonical NF-κB pathway [3].

In this study, we set out to disentangle the contribution of the various subunits in the maintenance and growth of CLL and MM cells by targeting the master regulator of the non-canonical signaling pathway, NF-κB-inducing kinase (NIK). Using a selective NIK inhibitor, CW15337 (Ki = 25 nM), with no inhibitory effects on IKKα or IKKβ at these concentrations [25], we showed that primary CLL cells as well as CLL and MM cell lines were all sensitive to the effects of NIK inhibition. Furthermore, the sensitivity of primary CLL samples to CW15337 was associated with the constitutive nuclear expression of the non-canonical NF-κB subunit, p52. This was also true for the CLL and MM cell lines, except for JJN3 cells, which possess an EFTUD2-NIK fusion gene, which lacks the TRAF3 binding domain resulting in the accumulation of a cytoplasmic EFTUD2-NIK fusion protein [4]. Three of the CLL samples tested were derived from patients with known mutations in BIRC3, that are associated with NIK stabilization and consequent p100 processing resulting in the nuclear translocation of p52. CLL patients with BIRC3 mutations experience a more rapid disease progression [13] and a poor response to fludarabine [30]. It is noteworthy that CLL cells derived from these patients showed increased nuclear p52 and high sensitivity to CW15337 (Figure 1E). The same samples were amongst the most resistant to in vitro challenge with fludarabine.

In accordance with previous reports, we showed that co-culture of MEC-1 cells and primary CLL cells induced proliferation and tumor cell activation (Figure 3E and Figure 4A). Furthermore, CD40L 3T3 co-culture markedly induced both canonical and non-canonical NF-κB signaling and the transcriptional activation of NF-κB target genes (Figure 4C and Figure 5A). The addition of CW15337 into the co-cultures inhibited cell growth and significantly reduced the amounts of nuclear p52 and RelB, which caused a concurrent decrease in the transcription of the anti-apoptotic BCL2-family genes, BCL2L1, MCL1 and to a lesser extent, BCL2A1 (Figure 5D–F). The CD40L-mediated increase in BCL2L1 (11.3-fold) and BCL2A1 (11.3-fold) transcription observed in our study were consistent with a previous report [25]. Furthermore, they showed that the BCL2L1 protein product, BCL-XL, was associated with CLL resistance to ABT-199, which was reversed by the addition of CW15337. In this study, we showed that MCL1 was also transcriptionally activated (1.5-fold) by co-culture; a finding at odds with one previous report [25], but corroborated by another [39]. We also showed a reduction in the transcription of BCL2A1 following exposure to CW15337, suggesting that BCL2A1 is, at least in part, transcriptionally regulated by non-canonical subunit activation. The reasons for these differences could include methodological discrepancies and/or possible variations in CD40L density on 3T3 cells. Importantly, both manuscripts concur regarding the importance of non-canonical NF-κB signaling as a driver of drug resistance in CLL and that BCL2L1 gene expression appears to play a critical role.

Here, we show that CW15337-mediated selective repression of non-canonical NF-κB subunits resulted in a strong synergy with the BCL2 antagonist, ABT-199 (venetoclax). A similar effect was observed when combining CW15337 with fludarabine. The mechanisms that underpin these positive drug interactions warrant further investigation but acquired drug resistance to venetoclax is commonly associated with the overexpression of BCL-XL and/or MCL1 [40,41]. Given that we demonstrated that CW15337 was able to repress the transcription of BCL2L1, BCL2A1 and MCL1, via the specific inhibition of non-canonical NF-κB subunits, it is tempting to consider that this repression is causal in the reversal of CD40L-mediated resistance to ABT-199 and fludarabine.

## 5. Conclusions

The data presented in this manuscript provide strong evidence that the inhibition of NIK, and the consequent repression of non-canonical NF-κB subunit activation, may be a useful therapeutic strategy. In particular, in targeting residual disease in the lymphoid and bone marrow niches of both CLL and MM, respectively. Given the concerns surrounding the safety of the systemic administration of drugs that target the canonical NF-κB pathway (IKKβ inhibitors) [3], we suggest that selectively targeting the non-canonical NF-κB pathway has the potential to reverse the acquired drug resistance frequently encountered when treating these two common B-cell malignancies.

## Figures and Tables

**Table 1:** Summary of CLL patient characteristics.

Parameter	Number
Number of CLL cases	15
Median age at sample collection (range)	64
	(48–80)
Gender	
Male	9
Female	6
Binet stage at diagnosis	
A	5
B	4
C	6
IGHV-mutated	7
IGHV-unmutated	8
CD49^neg^ (<30%)	6
CD49^pos^ (^3^30%)	9
CD38^neg^ (<20%)	7
CD38^pos^ (^3^20%)	8
B2M (<3.5mg/L)	5
B2M (^3^3.5 mg/L)	10
Chromosomal aberrations	3
11q-	1
17p-	9
13q-Trisomy 12	1
Genetic mutations	
TP53 mutation	1
BIRC3 mutation	3
NOTCH1 mutation	1
SF3B1 mutation	2

*IGHV*-mutated: >2% deviation from the germline immunoglobulin sequence; *IGHV*-unmutated: ≤2% deviation from the germline immunoglobulin sequence; B2M: beta 2 microglobulin; ND: not determined.

## Data Availability

The data generated in this publication will be deposited in NCBI’s Gene Expression Omnibus and will be accessible through GEO Series accession number GSE198456 (https://www.ncbi.nlm.nih.gov/geo/query/acc.cgi?acc=GSE198456, accessed on 30 January 2022).
